# Pluripotency Potential of Embryonic Stem Cell-Like Cells Derived from Mouse Testis 

**DOI:** 10.22074/cellj.2019.6068

**Published:** 2019-06-15

**Authors:** Hossein Azizi, Behruz Asgari, Thomas Skutella

**Affiliations:** 1 Faculty of Biotechnology, Amol University of Special Modern Technologies, Amol, Iran; 2 Department of Stem Cells and Developmental Biology, Cell Science Research Center, Royan Institute for Stem Cell Biology and Technology, ACECR, Tehran, Iran; 3 Institute for Anatomy and Cell Biology, Medical Faculty, Heidelberg University, Im Neuenheimer Feld, Heidelberg, Germany

**Keywords:** Mouse Testis, Pluripotency Potential, Spermatogonial Stem Cells

## Abstract

**Objective:**

During the cultivation of spermatogonial stem cells (SSCs) and their conversion into embryonic stem-like (ES-like) cells, transitional ES-like colonies and epiblast-like cells were observable. In the present experimental study, we aimed to analyze the efficiency of the multipotency or pluripotency potential of ES-like cells, transitional colonies and epiblast-like cells.

**Materials and Methods:**

In this experimental study, SSCs were isolated from transgenic octamer-binding transcription factor 4 (Oct4)-green fluorescent protein (GFP)-reporter mice. During cell culture ES-like, transitional and epiblast- like colonies developed spontaneously. The mRNA and protein expression of pluripotency markers were analyzed by Fluidigm real-time polymerase chain reaction (RT-PCR) and immunocytochemistry, respectively. Efficiency to produce chimera mice was evaluated after injection of ES and ES-like cells into blastocysts.

**Results:**

Microscopic analyses demonstrated that the expression of Oct4-GFP in ES-like cells was very strong, in epiblast-like cells was not detectable, and was only partial in transitional colonies. Fluidigm RT-PCR showed a higher expression of the germ cell markers Stra-8 and *Gpr-125* in ES-like cells and the pluripotency genes *Dppa5, Lin28, Klf4, Gdf3* and *Tdgf1* in ES-like colonies and embryonic stem cells (ESCs) compared to the epiblast-like and transitional colonies. No significant expression of *Oct-4, Nanog, Sox2* and *c-Myc* was observed in the different groups. We showed a high expression level of *Nanog* and *Klf4* in ES-like, while only a partial expression was observed in transitional colonies. We generated chimeric mice after blastocystic injection from ES and ES-like cells, but not from transitional colonies. We observed that the efficiency to produce chimeric mice in ES cells was more efficient (59%) in comparison to ES-like cells (22%).

**Conclusion:**

This new data provides more information on the pluripotency or multipotency potentials of testis-derived ES-like cells in comparison to transitional colonies and epiblast-like cells.

## Introduction

It is well known that mouse spermatogonial stem cells (SSCs) are unipotent stem cells, which express both pluripotency and germ cell markers (1-3). SCCs are capable of spontaneously transforming into pluripotent embryonic stem (ES)-like cells under germ cell culture conditions without the artificial addition of exogenous pluripotency genes or small molecules (1, 4, 5). These pluripotent ES-like cells in turn can convert into several cell lineages including the three embryonic germ layers and germ cells (1, 5-8).

Kanatsu-Shinohara et al. (5), produced ES-like cells within 4-7 weeks post-culture initiation in neonatal SSC cultures for the first time, and were followed by other groups, who showed that these pluripotent stem cells can be derived from the murine testis cells from up to 7-week old adolescent mice (1, 5). In the study by Kanatsu- Shinohara et al. (5), the ES-like cells played an active part in their transformation into germline chimeras following injection into blastocysts. These findings demonstrated the pluripotency of SSCs or neonatal testis-derived gonocytes; however, the derivation processes of ES-like cells from SSCs remained unclear. Other more studies have successfully generated different populations of Stra8-positive, GPR125-positive and Oct4-positive SSCs from ES-like cells (4, 6, 7). In a study by Guan et al. (7) multipotent ES-like cells could be derived from Stra8- positive SSCs from 7 week old mice, *in vitro*. Similar to the study by Kanatsu-Shinohara et al. (5) under *in vitro* and *in vivo* conditions, these cells could differentiate into all three germ layers *in vitro* and produced teratomas. After injection of Stra8-positive SSCs into blastocysts chimeras was formed (7). After mating, the chimera transmission to the next generation was observed. Germline transmission of Stra8-GFP-positive ES-like cells was not evaluated. Ko et al. (4) repeated the induction of pluripotency in 5-7 weeks Oct-4-GFP-positive adolescent SSCs. The authors described that the induction of differentiation dependends on the initial number of plated SSCs and the length of Oct4-positive cell culturing time without splitting. They manually picked the heterogonous Oct4-GFP-positive SSCs and demonstrated the relation between a certain number of SSCs (1000-4000) and a culture duration of 2-4 weeks for the induction of pluripotency. In a published protocol, this group described the conversion of SSCs into pluripotent stem cells only with SSCs of adolescent mice from postnatal day 35 (5 weeks old). The generated cells fulfilled the same criteria described by Kanatsu- Shinohara et al. (5) and Guan et al. (7). In another study this group generated ES-like cells from unselected testis cells of a testis biopsy (9). Seandel et al. (6) produced adult spermatogonial-derived stem cells from *GPR-125* LacZ-positive cells in 3-week to 8-month old mice, but these cells were only multipotent, because no germline transmission was observed in the chimera.

Although we have not come to a complete understanding of the reprogramming mechanism and the establishment of ES-like cells from SSCs, it is obvious that the reprogramming process is influenced by various conditions. These include the age of the donor animals the SSC plating density, the time period post-culture initiation, the culture duration, and the cell population variations observed while in culture (1, 5, 7, 10). Furthermore, during conversion to pluripotent cells different types of colonies can be observed, including ES-like cells, epiblast-like cells and semi-transmitted transitional colonies (1, 6, 7).

In the current study, the multipotency or pluripotency potentials of testis-derived ES-like cells, epiblast-like cells and transitional colonies were examined by using molecular characterizations and chimera assays in comparison to ESCs.

## Materials and Methods

### Isolation of embryonic stem-like cells, epiblast-like cells and transitional colonies

All animal care was performed according to guidelines of the Institute for Anatomy and Cell Biology of Heidelberg University (Heidelberg, Germany) and the Royan Institutional Review Board and Institutional Ethical Committee (Tehran, Iran). Testis cells were isolated from 4-week old C57BL/6 Oct4-promoter reporter GFP transgenic mice after decapsulation and treatment by a one-step enzymatic digestion protocol. Germline stem cells (GSCs) were established according to our previous study (1). The above-mentioned produced colonies were sub-cultured in mouse ES cell medium with KnockOut™ Dulbecco’s Modified Eagle’s Medium (KODMEM) or DMEM high-glucose medium (Invitrogen, USA), supplemented with 15% fetal bovine serum (FBS, Invitrogen, USA), 1% Non-Essential Amino Acid (NEAA) solution (Invitrogen, USA), 1% L-glutamine (Invitrogen, USA), 1% Pen-Strep (PAA) (Invitrogen, USA), 0 .1% β-mercaptoethanol (Invitrogen, USA) and leukemia inhibitory factor (LIF, Millipore, USA) at a final concentration of 1000 U/ml (1).

### Fluidigm biomark system gene expression analyses

The expression of various pluripotency- and germ cellassociated genes *Oct4, Nanog, Sox2, Klf4, c-Myc, Lin28, Gdf3, Tdgf1, Dppa-5, Stra8* and *Gpr-125* was analyzed utilizing dynamic array chips (Table 1). The housekeeping gene, *Gapdh*, was selected for normalization of data in different cultured cell types, including ESCs, ES-like cells, epiblast-like cells and transitional colonies. The expression fold change of mRNA was compared to mouse embryonic fibroblasts (MEF) feeder cells as an additional control. With the help of a micromanipulator (Narashige Instruments) about 50 cells were manually selected from each sample. Afterwards, the selected cells were lysed with a special lysis buffer containing 9 μl RT-PreAmp Master Mix (5.0 μl Cells Direct 2× Reaction Mix) (Invitrogen, USA), 2.5 μl 0.2× assay pool, 0.2 μl RT/Taq Superscript III (Invitrogen, USA) and 1.3 μl TE buffer and directly frozen and stored at -80˚C. The targeted transcripts were quantified with TaqMan real-time PCR on the BioMark real-time quantitative PCR (qPCR) system (Fluidigm, USA), with TaqMan gene expression assays (Invitrogen, USA) in 48.48 dynamic arrays. Two technical replicates were processed to analyze every sample. The CT values were analysed with GenEx software from MultiD, Excel and SPSS (1, 3, 11).

### Immunocytofluorescent staining

For immunocytochemistry each cell type was cultured in 24-well plates and fixated in 4% paraformaldehyde. After rinsing, the samples were permeabilized with 0.1% Triton/phosphate buffered saline (PBS, Sigma, USA) and unspecific staining sites were blocked with 1% bovine serum albumin (BSA)/PBS. The cells were incubated overnight with primary antibodies for Nanog (Abcam, USA) and Klf4 (Cell Signaling, USA). After rinsing several times with PBS, the cells were incubated with species-specific secondary antibodies conjugated to different fluorochromes. Afterwards, the stained cells were counterstained with DAPI (0.2 μg/ ml 4’, 6-diamidino-2-phenylindole) (Sigma, USA) for 3 minutes at room temperature and fixed with Mowiol 4-88 reagent (Sigma, USA). As a negative control for all antibodies, the omission of each primary antibody in the sample was performed. The labeled cells were examined with a confocal microscope (Zeiss LSM 700) and images were obtained using a Zeiss LSMTPMT (1, 2).

### Production of chimeric mice

The differentiation potentials of ES cells and ESlike cells *in vivo* was examined utilizing chimera generation. At 3.5 days post-coitus, blastocysts were harvested from super-ovulated female mice and placed in M2 medium. Subsequently, 10-15 single-cell colonies were transferred into each blastocyst. About 10 injected embryos were surgically transplanted into the uterine horns of pseudo-pregnant recipient female mice. The coat color of the chimera mice was used for their identification (1).

**Table 1:** List of the TaqMan gene expression assays for multiplex quantitative reverse transcription-polymerase chain reaction (qRTPCR)


Gene	Gene name	Species	Assay ID

*Dazl*	Deleted in azoospermia-like	Mouse	Mm00515630_m1
*Ddx4* or *Vasa*	DEAD (Asp-Glu-Ala-Asp) box polypeptide 4	Mouse	Mm00802445_m1
*Zbtb16* or *Plzf*	Zinc finger and BTB domain containing 16	Mouse	Mm01176868_m1
*Stra-8*	Stimulated by retinoic acid gene 8	Mouse	Mm01165142_m1
*Nanog*	Nanog homeobox	Mouse	Mm02384862_g1
*Lin28a*	Lin-28 homolog A (C. elegans)	Mouse	Mm00524077_m1
*Tdgf1*	Teratocarcinoma-derived growth factor 1	Mouse	Mm00783944_g1
*Dppa5*	Developmental pluripotency associated 5	Mouse	Mm01171664_g1
*Gdf3*	Growth differentiation factor 3	Mouse	Mm00433563_m1
*Pou5f1* or *Oct-4*	POU domain, class 5, transcription factor 1	Mouse	Mm03053917_g1
*Sox2*	SRY (sex determining region Y)-box 2	Mouse	Mm00488369_s1
*Gapdh*	Glyceraldehyde-3-phosphate dehydrogenase	Mouse	Mm99999915_g1


### Statistical analysis

The experiments were repeated at least three times. The average gene expression in each group was quantified, and One-way analysis of variance (ANOVA) followed by the Tukey’s post-hoc tests was employed to evaluate the experimental results.

## Results

### Characterization of embryonic stem-like cells, epiblast-like cells and transitional colonies

**Fig.1:**
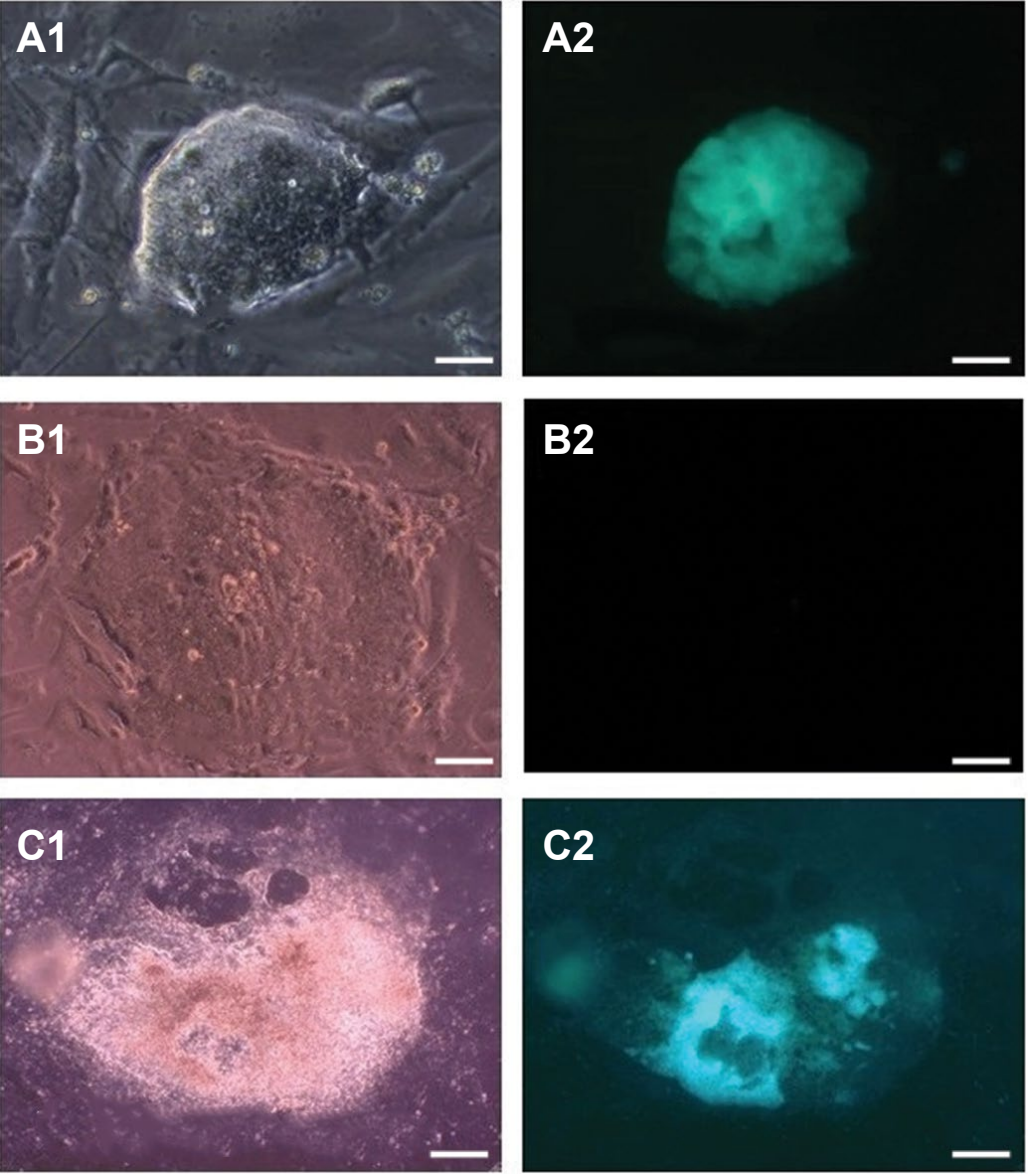
Different types of colonies are observed in spermatogonial stem cells (SSCs) cultures. Cell morphology and Oct4-GFP signals in **A1.** ESlike colonies, **B1.** Epiblast-like cells, **C1.** Transitional colonies, A2, B2, and **C2.** Show expression level of Oct4-GFP in the related cells (scale bar: 100 μm).

The characterization of the GSCs was established as described in our previous study (1). During passages of GSCs, we rarely found colonies which were similar to mouse ESCs that expressed high levels of Oct4-GFP, transitional colonies with partial expression of Oct4- GFP, or and epiblast-like cells without expression of Oct4-GFP. About two months after initiation of GSC cultivation, according to morphological criteria and the re-occurring Oct4-GFP reporter signal, ESlike colonies, epiblast-like colonies and transitional colonies were observed (Fig .1).

The ES-like colonies had a packed spindle- to round-shaped morphology with smooth borders and expressed the Oct4-GFP signal at a very high intensity throughout the whole area of the colonies (Fig .1A). In contrast, the epiblast-like cell colonies had a flat morphology with no expression of Oct4-GFP (Fig .1B). The transitional cell colonies were characterized by a jagged, irregular or uneven border with a partial expression of Oct4-GFP in some areas of the colonies (Fig .1C).

**Fig.2:**
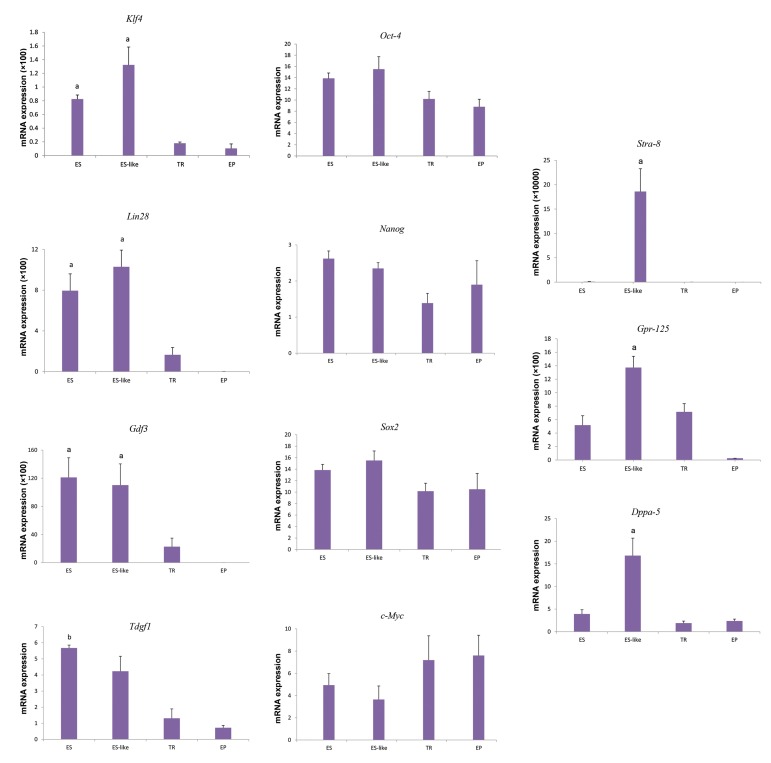
mRNA expression of pluripotency and germ cell genes. Analysis was performed comparing embryonic stem cells (ESCs), ES-like colonies, epiblast-like cells (EP) and transitional colonies (TR). Y-axis denotes fold change of mRNA expression in comparison to MEF feeder cells. Significance of the difference between the different groups was determined with t test. a; At least P<0.05 versus other groups, b; At least P<0.05 versus EP and TR cells, and MEF; Mouse embryonic fibroblasts.

In the next step, we examined the expression of pluripotency markers with Fluidigm RT-PCR for the ES cells, the ES-like, epiblast-like, and transitional colonies (Fig .2). The germ cell markers *Stra8* and *Gpr125* were expressed more strongly in the ESlike cells compared to any other group (P<0.05, Fig .2). Furthermore, higher expression levels of the pluripotency genes *Klf4, Gdf3* and *Lin28* were observed in ES-like cells and ESCs in comparison to the epiblast-like and transitional colonies (Fig .2). We also observed a significantly higher expression of *Tdgf1* in ESCs in comparison to the other groups (P<0.05, Fig .2). While the expression of *Dppa5* in the ES-like cells was significantly higher than in the other groups, we did not observe any significant difference in the expression of *Oct4, Nanog, Sox2* and *c-Myc* among the groups (Fig .2).

**Fig.3:**
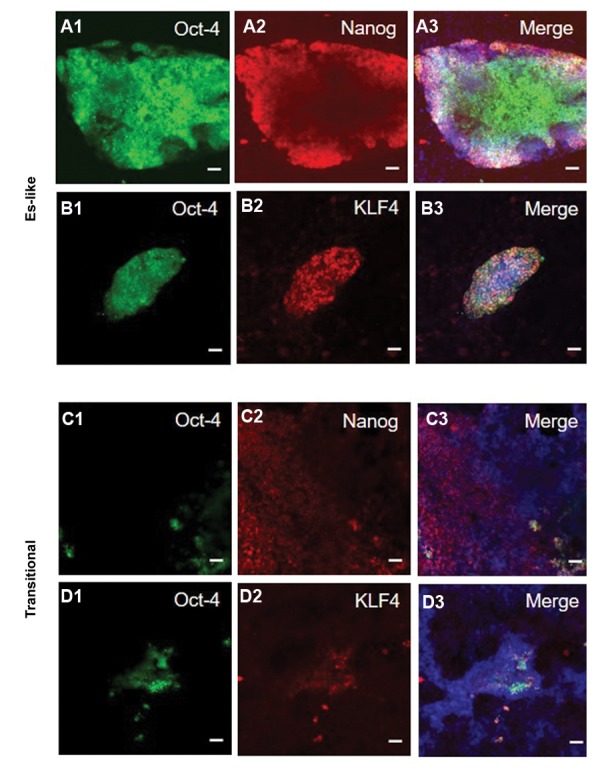
Immunocytochemical characterization of reprogrammed embryonic stem (ES)-like and transitional colonies. A1-A3. Oct4-GFP positive cells in the ES-like colonies are positive for the markers of Nanog, B1-B3. Klf4, while transitional colonies partially express Oct4-GFP signal, C1-C3. Nanog, and D1-D3. KLF4 (scale bar: 50 μm).

Furthermore, by immunocytochemistry we detected Oct4-GFP positive cells in the ES-like colonies that were strongly stained for Nanog (Fig .3A1-3), and KLF4 (Fig .3, Fig B1-3), while the transitional colonies only partially expressed Oct4-GFP and Nanog. Similar to the ES-like cells, in the transitional colonies there were areas that were partially positive for both Oct4- GFP and KLF4 (Fig .3).

### Chimeric mice production

In an additional experiment, we investigated the efficiency of the generation of chimeric mice with blastocyst injection of mouse ESCs and ES-like cells. We injected 164 embryos with ESCs and 169 embryos with ES-like cells that differentiated into blastocysts, which were implanted into a foster mother. We observed after embryo transfer 86 live births from ESCs and 41 from the ES-like group. The efficiency of the production of chimeric mice with ESCs was more efficient than the ES-like cells. Overall, 51 (59.3%) chimeric mice were generated after injection of ESCs and 9 (22%) chimeras from ES-like cells.

### 

## Discussion

In our study we demonstrated that ES-like, epiblast-like and transitional colonies all emerge during the conversion stage of unipotent GSCs into pluripotent cells, but only ES-like cells possess pluripotent stem cell potentials. Epiblast-like colonies are Oct4-GFP negative and are unable to shift to a pluripotent state. Transitional colonies are heterogeneous with only a partial expression of pluripotency genes. Only the strongly Oct4-GFP positive ES-like cells expressed a full network of pluripotency genes and contributed to the chimera assays. On the other hand, and in contrast to ESCs, the ES-like cells in parallel still strongly express the germ cell specific genes *Stra-8* and *Gpr-125*.

It is well documented that the transcriptional factor network of Oct-4, Nanog and Sox2 controls the pluripotency state in ESCs and is essential for the reprogramming of somatic cells into induced pluripotent stem cells (IPSCs) (12). This network blocks the expression of different lineage-specific genes and thus sustains pluripotency of the cells, preventing their differentiation (13). The level of expression of pluripotency gene is critical for the maintenance of pluripotency state (3, 14). In the colonies from the Oct4-GFP reporter mice, the GFP signal was strongly present only in the ES-like cells and partially in the transitional colonies. We confirmed that the expression of the pluripotency markers Nanog, and C-Myc in ES-like, transitional and epiblast-like cells was at similar levels in comparison to ESCs, while Sox2 was only strongly expressed in mouse ESCs. Our study also demonstrated partial and low expression levels of *Lin28* and *Klf4* in transitional and epiblast-like cells. The mRNA expression profiling confirmed that the expression levels of pluripotency markers were not the same, and significant differences were detected even between mouse ESCs and ES-like cells.

It has been suggested that the Oct4 protein, encoded by the *Pou5f1* gene, is absolutely required for the stemness properties of ESCs. During early embryonic development, Oct4 is expressed in blastomeres and in the inner cell mass (ICM) of the blastocysts, from which ESCs are derived *in vitro* (15, 16). After gastrulation, the Oct4 protein is down-regulated in the trophectoderm and in the primitive endoderm, but is maintained in primordial germ cells (PGCs). PGCs continue to express of Oct4 until the initiation of spermatogenesis in males or oogenesis in females (16). In concert with Oct4 and Sox2, the transcription factor Nanog is a key factor to establish ESC identity and to maintain pluripotency. Nanog seems to maintain self-renewal in mouse ESCs with an independent mechanism of the leukemia inhibitor factor (LIF)/signal transducer and activator of transcription 3 (Stat3) signalling pathway. The deletion of Nanog in mouse ESCs leads to a loss of the pluripotency state and induces differentiation into the extra-embryonic endoderm cell lineage (17). It has been shown that SSCs express all of the different Yamanaka factors (*Oct3/4, Sox2, Klf4, c-Myc*) at the mRNA level (11, 18), while mRNA for Sox2 is not translated into a protein (19). It has been documented that blastocysts with a deficiency in Sox2 lose their pluripotent state, therefore are unable to shape a pluripotent ICM (20). Klf4 has also been shown to be an important transcription factor for the regulation of pluripotency in cells (21). Klf4 accompanied by the genes *Oct4, Sox2* and *c-Myc*, is the main transcription factor for the generation of iPSCs from somatic cells (21, 22). Lin28 is a marker of ESCs and is expressed in undifferentiated mouse SSCs (23). Gillis et al. (24) reported that Lin28 might not be present in adult human testes, but they observed a high expression in human testicular germ cell tumors. We reported significantly high expression levels of Lin28 in mouse ES-like cells and ESCs compared to both epiblast-like and transitional cells.

There are diverse controversial challenges about the pluripotency and multipotency of ES-like cells (25- 28), including germ cell contribution and germ cell transmission.

Although it seems that ES-like cells from 4-week old Oct4-promoter reporter GFP transgenic mice have pluripotency potentials, in our experiments the efficiency for the production of chimeric mice from ESCs and ESlike cells was different. Our analysis showed that although chimeric mice could be generated in 22% of the cases after injection of ES-like cells into blastocysts, the efficiency to produce chimeric mice was lower than that after injection of ESCs (59%). Therefore, although ES-like cells express pluripotency markers and produce chimera, the degree of chimerism is not the same as the mouse ESCs.

In previous studies, Kanatsu-Shinohara et al. (5) microinjected ES-like cells from neonatal mice into blastocysts and observed chimerism in 36% (13 of 36) of the newborn animals as judged by EGFP-positive cells. According to their findings, donor cells were found in the central nervous system, liver, heart, lung, somites, intestine, and also in the germ cells in the testis of a 6-week old animal. Two offspring were obtained after performing microinsemination with EGFP-positive spermatids. By using the tetraploid complementation technique embryos, this group could generate embryos but no living offspring. This observation was explained by the altered imprinting status of the germ stem cells in comparison to mouse ES cells. Guan et al. (7) microinjected blastocysts with SSCs from 4 week old mice and detected chimaerism in 39 of 42 of mice (93%). After mating of chimeric males and females, these authors observed germline transmission. Furthermore, Ko et al. (4) performed chimera assays and observed germline transmission with mES-like cells at a lower level than with mouse ES cells.

Seandel et al. (6) generated GPR125 positive multipotent ES-like cells, which contributed to all three germ layers in embryoid body (EB) cultures and teratoma assays, but did not show germline transmission in chimeric embryos. Naive and prime pluripotency states have been demonstrated in pluripotent cells (29-32). Primary pluripotent stem cells, similar to late epiblast cells or postimplantation epiblast cells, could only produce chimeric animals to a limited extent (30). Therefore, it might be argued that the ES-like cells generated by Seandel et al. (6) were primary pluripotent cells (7).

To better understand the origin of the pluripotent stem cells, Guan et al. (33) generated ES-like cells from Stra8- GFP mice, while Seandel et al. (6) utilized GPR125 LacZ mice. However, the resulting cell lines were not from an initial germ cell population grown from a single cell (clonal growth) and from an ideal ES morphology (transitional morphology for Seandel) (4). Ko et al. (4) described clonal generation of ES-like cells, but the source of SSCs is unclear, because at a closer look Oct4- GFP SSCs lose the fluorescent signal after an initial germ cell culture (1, 4). Therefore, the ES-like cells, in which a strong GFP signal re-occurs, might have been generated from different types of SSCs or even other cells. In the past years, the enhancement of ES-like cell production by certain chemicals has been proven. The derivation of pluripotent ES-like cells could be expanded by glycogen synthase kinase-3 inhibition in neonate mouse testicular cultures (28).

## Conclusion

During the culture of mouse testicular GSCs from 4-7 week old mice, different types of colonies are spontaneously generated, while only ES-like colonies are able to reach a full pluripotent state. The different types of colonies can be distinguished by morphological criteria. Only ES-like and transitional colonies show Oc4-GFP reporter signals, “epiblast like” colonies are Oct4-GFP negative. In contrast to ESCs, Oct4-GFP positive ES-like cells still express the germ cell specific genes Stra8 and GPR125, indicating that the Oct4-GFP positive ES-like cells maintaine their original epigenetic "germ cell memory". The remaining epigenetic germ cell-associated traces have to be further researched in the future. This observation might also be interesting for the generation of germ cells from pluripotent ES-like cells. The efficiency to produce chimeric mice was more efficient with mouse ESCs in comparison to ES-like cells. Further research of chimeric mouse production by ES-like cells has to be conducted in higher numbers, while analyzing the potency of these cells for germline transmission in more detail.
